# The Treatment of Superfluous Hairs

**Published:** 1899-06-03

**Authors:** 


### THE TREATMENT OF SUPERFLUOUS HAIRS.

Some correspondence which has lately taken place in the *Lancet* in regard to the best treatment of superfluous hair shows how greatly this must depend upon the age of the patient, and the thickness with which the hairs are planted. The removal of thick but more or less solitary hairs by means of electrolysis is a comparatively simple matter. It must be remembered, however, that, although, if the operation is properly performed, the scar produced by the proceeding is but a small one, still there is a minute white cicatrix which in the end becomes somewhat depressed, and although such a cicatrix standing by itself is a matter of indifference, it is otherwise when a few hundreds of such scars are closely packed, as is necessarily the case when the attempt is made to remove a moustache by this means. In such case the whole structure of the skin over the affected area becomes so permeated by lines of cicatricial tissue as to appear white, atrophied, and scar like, a condition far more offensive to most eyes than even a pronounced moustache. The correspondence arose in regard to the treatment of a young lady, 26 years of age, of great beauty, but unfortunately afflicted with a moustache of a dark colour which was gradually growing, the hairs having already reached an eighth of an inch in length. Those correspondents who appear to know most about the subject are unanimous in saying that whatever else is done electrolysis should not be employed in such a case, the remedy being distinctly worse than the disease.

The most original and useful suggestion comes from Dr. Ethel Williams, who, arguing that it is chiefly by virtue of the darkness of the hairs that the moustache becomes an eyesore, advises that it should be bleached. In dealing with such cases she orders a lotion of peroxide of hydrogen, to be applied to the part every three or four nights. and the hairs well soaked in it. The hairs are thus rendered colourless and practically invisible. Of course, the treatment has to be continued at invervals, but we are assured that it is not distasteful to the patient in the way that regular shaving is. Dr. Balamno Squire, commenting on this method, asks whether the peroxide when applied habitually, in sufficient strength and for sufficiently long to completely bleach a black feminine moustache, is found to irritate the skin of the upper lip, more especially at the angles of the mouth, for he has found that the habitual use of depilatories to the upper lip is apt to make the skin at the angle of the mouth very raw indeed; and that even the use of pumice stone, which is a favourite plan with many women, is liable to have the same effect. To this Dr. Ethel Williams answers that she has found that to reduce the hairs to a pale golden colour is sufficient to make them quite unnoticeable. The applications must be thorough and repeated; and, although the hair under this treatment becomes very brittle and splits and breaks a good deal—a fact which ladies who think to produce golden tresses by the use of this agent may well bear in mind—this may be looked upon as rather an advantage in the case in question. As to the effect on the skin, her experiments have shown that a certain amount of irritation is produced, but not more than quite disappears in a few hours with a good wash and a little vaseline.

The question arises why do young women who are troubled in this way object to the use of the razor? Dr. Squire suggests that a girl so situated shrinks from being a fraud to the man she may marry. Her view is that if she can get her moustache permanently removed by some device or another, whatever may have been the case in the past she is what she seems to be, namely, a woman without a moustache; whereas if she only keeps it down by regular shaving she remains a fraud, and some men might find a difficulty in reconciling themselves to the discovery. In addition to this, however, the objection to shaving is no doubt to some extent due to the very prevalent idea that cutting will make the hair grow more thickly, coarser and longer. As to this, Dr. Squire says that he has satisfied himself by careful and repeated experiment that such results do not follow. Pulling the hairs out over and over again will make them grow much coarser, but not cutting or shaving. In the treatment of senile hirsuteness the problem is rather different. There the hairs of the moustache are long and coarse, and are spaced much more widely apart, and in such cases treatment by electrolysis is capable of producing a satisfactory result.

